# Immune-infiltrating signature-based classification reveals CD103^+^CD39^+^ T cells associate with colorectal cancer prognosis and response to immunotherapy

**DOI:** 10.3389/fimmu.2022.1011590

**Published:** 2022-10-12

**Authors:** Yang Luo, Yunfeng Zong, Hanju Hua, Meiting Gong, Qiao Peng, Chen Li, Dante Neculai, Xun Zeng

**Affiliations:** ^1^ State Key Laboratory for Diagnosis and Treatment of Infectious Diseases, National Clinical Research Center for Infectious Diseases, National Medical Center for Infectious Diseases, Collaborative Innovation Center for Diagnosis and Treatment of Infectious Diseases, The First Affiliated Hospital, Zhejiang University School of Medicine, Hangzhou, China; ^2^ Department of Cell Biology, Department of Pathology Sir Run Run Shaw Hospital, Zhejiang University School of Medicine, Hangzhou, China; ^3^ Research Units of Infectious disease and Microecology, Chinese Academy of Medical Sciences, Hangzhou, China; ^4^ Colorectal Surgery Department, The First Affiliated Hospital, Zhejiang University School of Medicine, Hangzhou, China; ^5^ Zhejiang University-University of Edinburgh Institute (ZJU-UoE Institute), Zhejiang University School of Medicine, Zhejiang University, Haining, China; ^6^ Department of Human Genetics, Women’s Hospital, Zhejiang University School of Medicine, Hangzhou, China; ^7^ Alibaba-Zhejiang University Joint Research Center of Future Digital Healthcare, Hangzhou, China

**Keywords:** colorectal cancer, immunotherapy, prognosis, high-dimensional single-cell analysis, immune cell diversity

## Abstract

**Background:**

Current stratification systems for tumor prognostic prediction and immunotherapeutic efficacy evaluation are less satisfying in colorectal cancer (CRC). As infiltrating immune cells in tumor microenvironment (TME) played a key role in tumor progression and responses to immune checkpoint blockade (ICB) therapy, we want to construct an immune-related scoring system with detailed immune profiles to stratify CRC patients.

**Methods:**

We developed a scoring system based on immune-related signatures and validated its ability to predict prognosis and immunotherapeutic outcomes in CRC. CD45^+^ cells from CRC patients were sorted to investigate detailed immune profiles of the stratification system using mass cytometry. A single-cell RNA sequencing dataset was used to analyze transcriptomic profiles.

**Results:**

We constructed an immune-related signature score (IRScore) based on 54 recurrence-free survival (RFS)-related immune signatures to stratify CRC patients. We revealed that IRScore was positively correlated with RFS and favorable outcomes in ICB treatment. Moreover, we depicted a detailed immune profile in TME using mass cytometry and identified that CD103^+^CD39^+^ T cells, characterized by an exhaustive, cytotoxic and proliferative phenotype, were enriched in CRC patients with high IRScore. As a beneficial immune signature, CD103^+^CD39^+^ T cells could predict prognosis and responses to ICB therapy in CRC.

**Conclusions:**

All the analyses above revealed that IRScore could be a valuable tool for predicting prognosis and facilitating the development of new therapeutic strategies in CRC, and CD103^+^CD39^+^ T cells were one of defined immune signatures in IRScore, which might be a key factor for antitumor immunity.

## Introduction

Colorectal cancer (CRC) ranks the third most commonly diagnosed malignant human cancer and the second leading cause of death worldwide (1). In China, 592,232 new colorectal cases and 309,114 CRC-related deaths were estimated to occur in 2022 (2). Currently, main therapeutic methods of CRC usually involve local treatments, including surgery removal, radiation therapy and systematic therapies like chemotherapy and targeted therapy. The most widely used CRC stratification system is the AJCC/TNM staging system (3). However, due to the significant heterogeneity in patients with CRC identified by the genetic or epigenetic investigation and transcriptomic profiles, patients with the same tumor stage may respond differently to the same treatment and thus lead to varying clinical outcomes. Therefore, there is a clinical requirement to establish a method for predicting CRC prognosis and evaluating the efficacy of immunotherapy.

Since its successful application in melanoma, tumor immunotherapy, especially immune checkpoint blockade (ICB), has been increasingly becoming a preferred consideration for various cancers (4–7). ICB treatment can reactivate exhausted functional cells and elicit durable antitumor responses. In this scenario, immune cells in tumor microenvironment (TME) play a key role in tumor progression and can influence the efficiency of ICB therapy. A positive response to ICB therapy relies on the context of TME and its interactions with tumor cells (8). TME is a complex and heterogeneous mixture of tumor cells, non-tumor cells such as infiltrating lymphocytes, macrophages, fibroblasts, stromal cells and other surrounding host cells, and non-cellular components like extracellular matrix (ECM) and secreted factors (9–12). These immune and non-immune cells with distinct functions can suppress or promote tumor progression. For example, higher level of cytotoxic T lymphocytes (CTLs) infiltration has been shown to be positively correlated with better antitumor responses and prognosis (13, 14). Thus, depicting the detailed infiltrating immune profiles and understanding the role of each immune subset in TME are required to improve the efficacy of ICB therapy. On the other hand, due to the complexity of TME, most patients, including CRC, do not benefit from current ICB therapy strategies, resulting in wasted healthcare resources and poor prognosis. Therefore, an effective stratification system, which can distinguish responders and non-responders for ICB therapy, can facilitate precision and personalized medicine. Since the infiltrating immune cells in TME played a key role in tumor prognosis and immunotherapeutic outcomes, an infiltrating immune cell signature-based subtyping system will be useful for tumor prognosis prediction and immunotherapeutic efficacy evaluation in CRC.

This study focused on immune-related signatures in TME and investigated the relationship between immune signatures and prognosis, and the association of immune signatures with response to ICB therapy in CRC. We evaluated 151 immune-related signatures in TME collected from public studies in 7 CRC cohorts and developed immune-related signature score (IRScore) based on 54 identified prognosis-related signatures. We depicted a detailed immune profile of IRScore by performing cytometry by time of flight (CyTOF) analyses of tumor infiltrating lymphocytes (TILs) in CRC. We revealed that CD103^+^CD39^+^ T cells were one of defined immune signatures in IRScore, and the CD45^+^CD3^+^CD103^+^CD39^+^ signature could predict patients’ prognosis and responses to ICB therapy (**Figure S1A**). Our study provides a useful tool for predicting CRC prognosis and response to ICB therapy and uncovers important clues to key immune subsets that affect tumor progression and outcomes of ICB therapy.

## Materials and methods

### Acquisition and pre-processing of CRC cohorts

Gene expression data and clinical information of CRC cohorts used in this study were acquired from UCSC Xena (15) and Gene Expression Omnibus (GEO) (16). Two CRC single-cell datasets were downloaded from GEO under the accession number GSE178341 and GSE108989. The metastatic urothelial cancer treated with anti-PD-L1 agent (atezolizumab) cohort IMvigor210 was obtained *via* R package *IMvigor210CoreBiologies* (17). The melanoma cohort undergoing anti-PD-1 checkpoint inhibition therapy was downloaded from GEO under accession number GSE78220. Only primary tumors and treatment-naive patients were included.

Microarray data (GSE103479, GSE17538, GSE33113, GSE37892, GSE38832, GSE39084 and GSE39582 used to develop IRScore; GSE39395 and GSE39396 used to draw signatures of fibroblasts, endothelial cells and epithelial cells) were downloaded and processed using the R package *GEOquery* (18). For TCGA cohort, log-transformed (on a base 2 scale) gene expression data were downloaded from UCSC Xena (https://xenabrowser.net/datapages/). Processed expression data of GSE178341, GSE108989 and GSE78220 were directly downloaded from GEO. Counts data and patients’ information from the IMvigor210 cohort were obtained by function *counts* and *pData*, and the gene counts were transformed into TPM for the following analysis. All datasets used in this study were listed in **Table S1**.

### Collection of immune-related signatures

One hundred forty-eight immune-related signatures were collected from previously published studies through a literature search (**Table S2**) (19–22). The signature genes of fibroblasts, endothelial cells and epithelial cells were obtained by performing differential analysis in GEO cohorts GSE39395 (immune cells: CD45^+^Epcam^-^, epithelial cells: CD45^-^Epcam^+^, stromal cells: CD45^-^Epcam^-^) and GSE39396 (immune cells: CD45^+^EPCAM^-^CD31^-^FAP^-^, epithelial cells: CD45^-^EPCAM^+^CD31^-^FAP^-^, endothelial cells: CD45^-^EPCAM^-^CD31^+^FAP^-^, cancer-associated fibroblasts: CD45^-^EPCAM^-^CD31^-^FAP^+^).

### IRScore calculation

First, we calculated a single sample gene set enrichment score for each patient using the *gsva* function implemented in the R package *GSVA* (23) and scaled the enrichment score to draw a normalized enrichment score (NES). Patients were divided into high and low groups according to the median value. Then Univariate Cox regression was performed to examine the relationship between NES and RFS in each CRC cohort. A meta-analysis implemented in the R package *meta* (24) was used to evaluate the Hazard Ratio (HR) and *P*-value. Only signatures with a *P*-value less than 0.05 were included. Totally we identified 54 immune-related signatures and classified them into 30 prognostically good signatures (HR< 1) and 24 prognostically bad signatures (HR > 1). We thus defined and calculated a so-called IRScore for each sample as:


$$\text{IRScore}=\sum_{i=1}^{M}NES_{i}-\;\sum_{j=1}^{N}NES_{j}$$

where *NESi* represents *NES* of *i*th prognostically good signature and *NESj* is *NES* of *j*th prognostically bad signature; *M* and *N* denote the number of prognostically good and bad signatures, respectively.

### Prediction of immunotherapeutic response

Prediction of ICB therapy response for TCGA and GSE39582 cohorts was conducted using the subclass mapping method (SubMap) (25). The SubMap module implemented in GenePattern (https://www.genepattern.org/) was used to conduct the prediction. Besides, a melanoma cohort treated with sequential CTLA-4 and PD-1 blockade was used to help predict patients’ responses to anti-CTLA-4 and anti-PD-1 treatment (26).

### Differential gene expression analysis and gene set enrichment analysis

Differential gene expression analysis for microarray data was performed by R package *limma* (27), and for TCGA counts data, *DESeq2* (28) was introduced for analysis. Gene set enrichment analysis and KEGG analysis was performed by R package *clusterProfiler* (29). HALLMARK gene sets and KEGG gene sets used in GSEA analysis were downloaded from the Molecular Signatures Database (MSigDB) (30). The curated signatures were obtained and summarized from previously published studies and provided in **Table S3** (31–34).

### Single-cell RNA-seq data analysis

Following the standard analysis procedures, scRNA-seq data were analyzed using the R package *Seurat* (35). For scRNA-seq data downloaded from GSE178341, 64 clusters were obtained using function *FindClusters*. IRScore was calculated for each cluster and each patient based on average expression data derived by the *AverageExpression* function. Clusters and patients were classified into high and low IRScore groups respectively. As for scRNA-seq data of CRC T cells from GSE108989, we calculated IRScore for each cell and likewise classified them into high and low IRScore groups according to the median value. UMAP and tSNE algorithms implemented in *Seurat* were used to visualize high-dimensional data.

### Bulk RNA sequencing and CyTOF

Thirteen fresh CRC tumor samples were collected from the First Affiliated Hospital, Zhejiang University School of Medicine. Clinical information of CRC patients was recorded in **Table S4**. All participants, or their legally authorized representatives, provided written informed consent upon enrollment. Each CRC tumor tissue was divided into two parts, one for RNA-seq and one for CyTOF. Tumor tissues were kept in RNAlater and total RNA was extracted using TRIzol reagent (Thermo Fisher Scientific). RNA-seq was performed at Beijing Genomics Institute (BGI) (Shenzhen, Guangdong, China) using the DNBSEQ system. UCSC hg38 reference genome was used to map the paired-end transcriptome reads. FPKM and read counts were generated for subsequent analysis.

The rest of tumor tissues from the same patients were transferred to MACS Tissue Storage Solution (Miltenyi Biotec), digested and prepared into single-cell suspensions as previously reported (36). Briefly, Samples were washed in RPMI 1640 (Thermo Fisher Scientific), suspended in 5 ml Hank’s Balanced Salt Solution (HBSS) (Thermo Fisher Scientific) with 1 μM DTT, 5 mM EDTA and incubated at 37°C shaker at 145 rpm for 30 minutes. After washing twice with RPMI 1640, samples were then mechanically dissociated with a sterile scalpel and digesting in a buffer cocktail containing 2 mg/ml collagenase IV (Sigma), 20 μg/ml DNase (Sigma) in RPMI 1640 for 2 hours in a 37°C shaker at 145 rpm in gentleMACS C tubes (Miltenyi Biotec), followed by dissociating on the gentleMACS Dissociator (Miltenyi Biotec) for 30 minutes. Tissue samples were filtered through a 100 μM cell strainer, washed, and enriched using 36% Percoll (GE Healthcare) at 2000 rpm for 10 minutes. Single cell suspensions were washed twice with PBS and stained with 5 μM 103Rh (Fluidigm) for 5 minutes at RT for viability. Cells were fixed in Fix I buffer (Fluidigm) at RT for 10 minutes and resuspended in freezing solution (90% FBS, 10% DMSO) after washing, and were stored at -80 ˚C for future use. A mass cytometry panel with 41 metal isotope-tagged antibodies (**Table S5**) was used to profile immune signatures in CRC samples. CyTOF was performed at Zhejiang Puluoting Health Technology Co., Ltd (Hangzhou, Zhejiang, China) by Helios (Fluidigm) with 300 events/s. Data were exported as FCS files. Fcs files were read into R by *read.FCS* function and signal intensities were arcsinh transformed with a cofactor of 5. The R function *metaClustering_consensus* implemented in package *FlowSOM* (37) was used to cluster all cells into 36 clusters. The *tSNE* algorithm was performed on 13,000 randomly selected cells (1000 cells per sample) to demonstrate high-dimensional data. The 99th percentile of maker intensity was defined as the maximum to exclude extreme value, and then all markers’ intensities were rescaled ranging between 0 to 1. The cluster-marker expression heatmap was generated by the R package *pheatmap* based on the median expression value.

### Statistical analyses

Log-rank test implemented in R package survival was used to evaluate differences in recurrence-free survival between high and low groups. The Kaplan-Meier curves were drawn using the R package *survminer*. R function *coxph* implemented in *survival* was used to compute the Cox proportional hazards regression model. The circular heatmap was visualized by R package *circlize* (38). Spearman’s correlation was calculated by function *rcorr* implemented in R package *Hmisc*. All data except mentioned above were displayed using R package *ggplot2* (39). Wilcoxon signed-rank test was used to compare the difference between two groups, and the Kruskal-Wallis test was used to compare differences among three or more groups. All analyses were conducted using R software (version 4.1.1). *P*-value< 0.05 was considered statistically significant unless explicitly noted.

## Results

### Construction of IRScore and the association with clinical and molecular phenotypes

**Figure 1:**
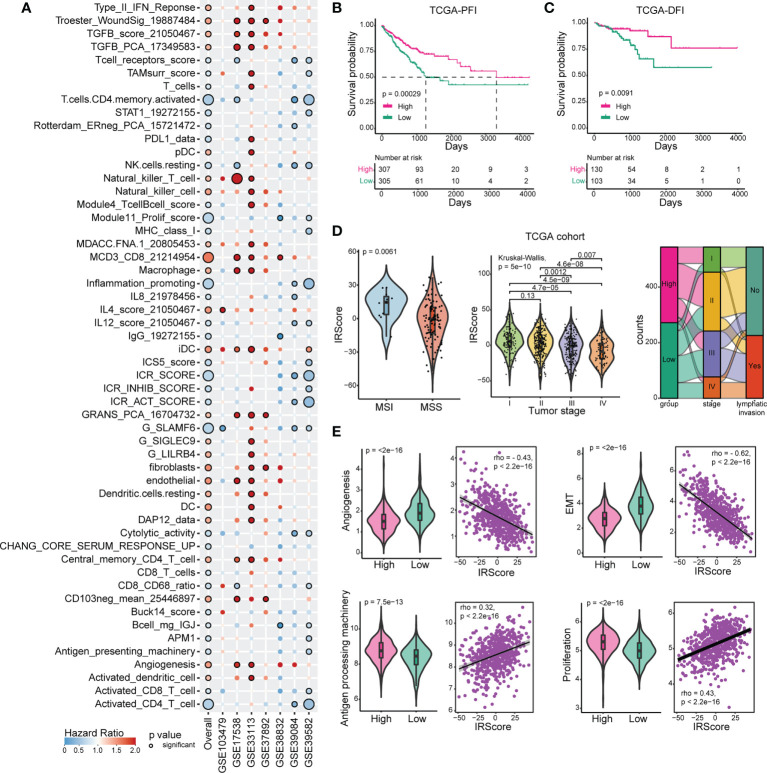
Construction and exploration of IRScore in CRC cohorts. **(A)**. Immune-related signatures with survival significance in CRC. Circles with black border represent prognostic significance of the signature in corresponding cohort, and the size of circles represents the significance level. Red circles represent Hazard Ratio (HR) >1, implying prognostically “bad” signatures, and blue circles represent HR< 1, implying prognostically “good” signatures. **(B, C)** Kaplan-Meier curves of progression-free interval (PFI, **B**) and disease-free interval (DFI, **C**) based on IRScore groups in TCGA cohort. **(D)**. Violin plots showing the relationship between IRScore and MSI/MSS (left), tumor stages (middle), and Sankey diagram illustrating the relationship between IRScore and CRC subtypes in TCGA cohort.**(E)**. The association and correlation between IRScore and gene signatures (angiogenesis, EMT, antigen presenting machinery and proliferation) in TCGA cohort illustrated by violin plots (left) and scatter plots with trend lines (right) in TCGA cohort.

To develop a predictive scoring system of recurrence-free survival (RFS) for CRC, single sample gene set enrichment analysis (ssGSEA) was performed to calculate enrichment score for each patient from 7 CRC cohorts based on 151 curated immune-related signatures. A univariate Cox regression model was applied to evaluate the predictive value of normalized enrichment score (NES) in each cohort. After leveraging 7 CRC cohorts, 54 immune-related signatures significantly associated with prognosis were identified (*P*-value< 0.05) (**Figure 1A**). We calculated IRScore for each patient in each cohort and stratified patients into high IRScore group and low IRScore group according to the median value, that is, CRC patients with IRScore higher than the median value were allocated as high IRScore group, and those lower than the median value as low IRScore group. Survival analysis revealed that patients in the high IRScore group had longer survival time without recurrence than those in the low IRScore group (**Figure S1B**). Same phenomena were observed in the combined dataset and in TCGA cohort regardless of progression-free interval (PFI), disease-free interval (DFI), disease-specific survival (DSS) or overall survival (OS) (**Figures 1B, C**, **S1B-E**).

Then we investigated the relationships between IRScore and clinical features. Non-metastatic CRCs with microsatellite instability/deficient mismatch repair (MSI/dMMR) had been reported to have better prognosis and immunotherapeutic outcomes than microsatellite stability/proficient mismatch repair (MSS/pMMR) (40). We revealed that CRC patients harboring MSI/dMMR had significantly higher IRScore than those with MSS/pMMR. IRScore differed among four summarized AJCC stages, with higher IRScore in early-stage patients and lower IRScore in advanced-stage patients, indicating that IRScore might be associated with tumor progression in CRC. The imbalanced components of high and low IRScore groups in tumor stages, lymphatic invasion and MMR were summarized by Sankey diagrams (**Figures 1D**, **S1F**). We also explored associations between IRScore and different molecular signatures. We observed that IRScore was negatively correlated with angiogenesis, epithelial-mesenchymal transition (EMT), and positively correlated with antigen processing machinery and proliferation (**Figure 1E**). Moreover, IRScore was positively associated with immune signatures such as CD8 T effector, cytotoxicity, immune checkpoint, proliferation-related signatures, and negatively associated with pan-fibroblast TGF-β response signature (pan-F-TBRS), naiveness and plasminogen inhibitor (**Figure S1G**). These data suggested that IRScore was positively correlated with factors for good prognosis in CRC.

### IRScore could be an indicator to predict responses to ICB therapy

The efficacy of ICB therapy targeting programmed cell death 1 (PD-1) or cytotoxic T-lymphocyte associated protein 4 (CTLA-4) was limited in CRC, so we examined the predictive and immunotherapeutic efficacy of IRScore. We used SubMap and a melanoma cohort treated with sequential CTLA-4 and PD-1 blockade to predict patients’ responses. Patients in the high IRScore group might respond to PD-1 inhibitors in both cohorts, while there was a possibility that patients in the low IRScore group in GSE39582 were likely to respond to CTLA-4 inhibitors (**Figures S2A, B**).

**Figure 2:**
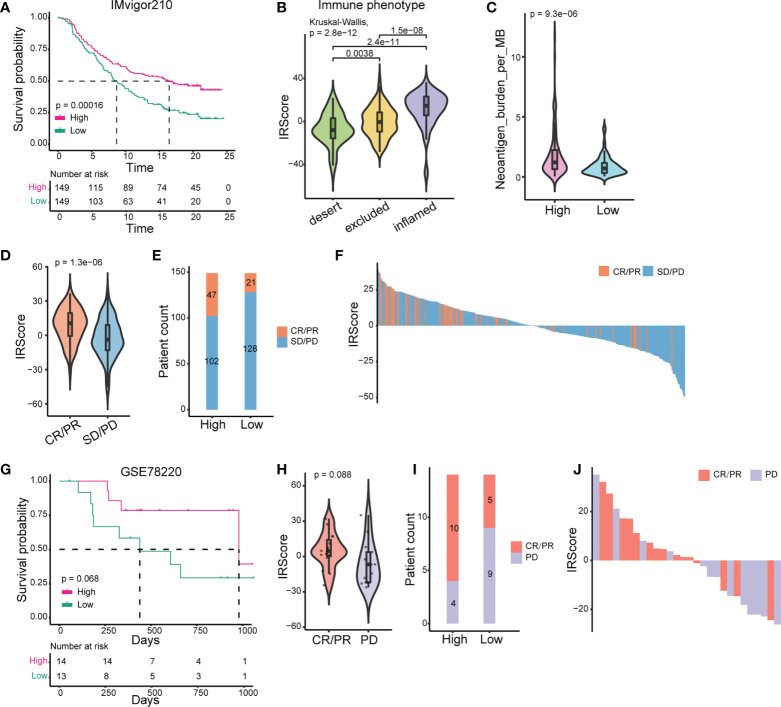
Prediction of patients’ responses to immunotherapy by IRScore. **(A)** Kaplan-Meier curve of survival probability based on IRScore groups in IMvigor210 cohort. **(B)** Comparison of IRScore among different immune phenotypes. **(C)** Comparison of neoantigen burden between high and low IRScore groups. **(D)** Violin plots of IRScore between CR/PR and SD/PD. **(E)** Compositions of patients’ responses to PD-L1 inhibitor treatment between high and low IRScore groups (P-value< 0.05, Fisher’s exact test). **(F)** Waterfall plots illustrating IRScore according to immunotherapeutic responses in IMvigor210 cohort. **(G)** Kaplan-Meier curve of survival probability based on IRScore groups in GSE78220 cohort. **(H)** Violin plot of IRScore between CR/PR and PD. **(I)** Compositions of patients’ responses to PD-1 inhibitor treatment in the two IRScore groups. **(J)** Waterfall plot illustrating IRScore according to immunotherapeutic responses in GSE78220 cohort.

We further used the published IMvigor210 cohort to investigate the predictive efficacy of IRScore. Patients with high IRScore significantly had longer survival time than those with low IRScore (**Figure 2A**). The inflamed phenotype presented the highest IRScore than desert or excluded phenotypes, and tumors in the high IRScore group had higher neoantigen burdens (**Figures 2B, C**). The results showed that the CR/PR group had the highest IRScore and patients in the high IRScore group displayed better responses to ICB (**Figures 2D-F**). The predictive efficacy of IRScore was also testified in GSE78220 cohort. High IRScore patients presented favorable responses and prolonged survival (**Figures 2G-J**). Together, these results implied that higher IRScore was associated with better responses and longer survival time in ICB treatment patients.

### Transcriptomic, genomic and immune signatures of high and low IRScore groups in CRC

**Figure 3:**
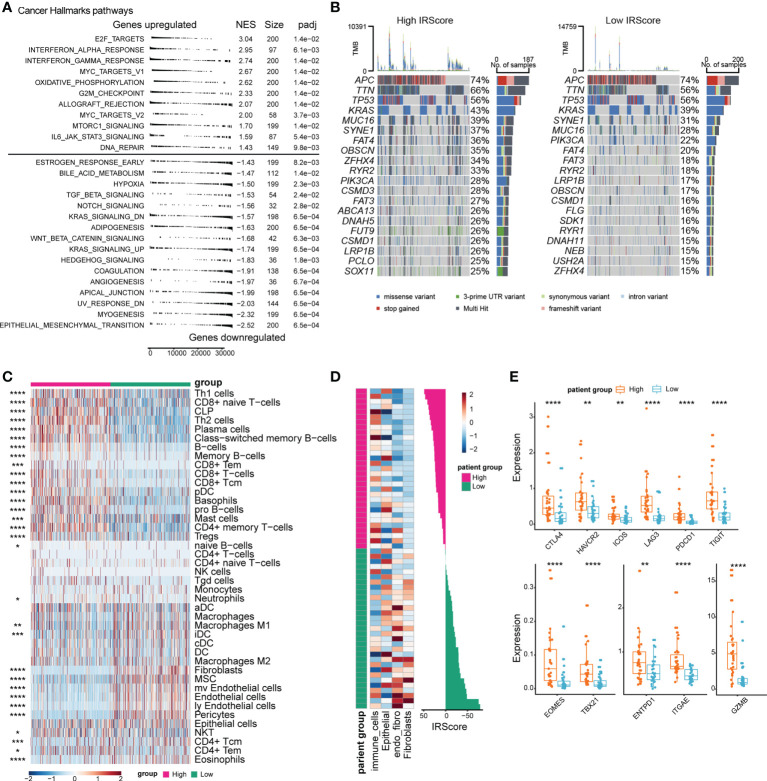
Comparison of transcriptomic profile, genomic alteration and immune infiltration between high and low IRScore groups. **(A)** Gene set enrichment analysis of Cancer Hallmark gene sets identified significantly enriched pathways in high and low IRScore groups. **(B)** Oncoplot showing the top 20 most frequently mutated genes in high and low IRScore groups in TCGA cohort. **(C)** Abundance of different cell types in high and low IRScore groups was estimated by xCell in TCGA cohort. Z-scored results were depicted in heatmap. **(D)** Abundance of different cell types in high and low IRScore groups in the single-cell dataset GSE178341. Z-scored results were depicted in heatmap (left). Waterfall plot illustrating IRScore of each patient according to high and low groups (right). **(E)** Box plots showing the expression level of indicated genes in the two IRScore groups. *P < 0.05, **P < 0.01, ***P < 0.001,****P < 0.0001.

Underlying changes accompanied the phenotypic differences between high and low IRScore groups. We assessed transcriptomic, genomic and immune features between high and low IRScore groups to investigate such changes. We first examined Cancer Hallmark gene sets in the two groups by GSEA. Gene sets related to proliferation and inflammation significantly contributed to the positive side, indicating that they were significantly enriched in up-regulated genes when comparing the high IRScore group to the low IRScore group. Epithelial-mesenchymal transition (EMT) and signaling pathways known to induce EMT, including TGF-β, Notch, Wnt-β-catenin and Hedgehog, were enriched in low IRScore tumors (**Figures 3A**, **S3A**). We further analyzed differentially expressed genes (DEGs, absolute log2FoldChange > 1, adjusted *P*-value< 0.05) between the two groups coupled with Kyoto Encyclopedia of Genes and Genomes (KEGG) pathways enrichment analysis. Consistently, the most enriched KEGG pathway represented by low IRScore tumors was ECM-receptor interaction. In high IRScore tumors, up-expressed genes were enriched for immune-related pathways implicated in processes crucial for host innate/adaptive immune responses (**Figures S3A, B**). Moreover, we analyzed gene mutation conditions of the two groups in TCGA cohort. The top 6 mutated genes were identical in the two groups but with distinct mutation frequencies except for APC (74%) and TP53 (56%). Besides, most genes tended to have higher mutation frequencies in the high IRScore group, in tune with the result that tumors with high IRScore had higher mutation burdens (**Figures 3B**, **S3C**).

Next, we investigated the TME characteristics of the two groups. Using xCell (41), we estimated immune and stromal cell compositions for patients with different IRScore in TCGA cohort. The high IRScore group was enriched with several CD8^+^ T cell types, Type 1 T helper (Th1), Type 2 T helper (Th2), B cell types, plasma cells, plasmacytoid dendritic cells (pDCs), and mast cells, but less fibroblasts, mesenchymal stem cells (MSCs), endothelial cells, pericytes (**Figure 3C**). To evaluate whether tumor-infiltrating cells displayed similar patterns as in TCGA cohort in a single-cell perspective, we utilized a single cell dataset (GSE178341) and classified tumor cells into 64 clusters. We identified 22 immune cell clusters, 33 epithelial clusters, 3 fibroblast clusters, 4 clusters expressing both endothelial and fibroblast markers (referred to as endo_fibro clusters) and 2 clusters expressing both epithelial and immune cell markers (referred to as other) (**Figures S3D, E**). We calculated cluster-level IRScore and classified 64 clusters into high and low IRScore groups. The high IRScore group consisted of 16 immune cell clusters, 15 epithelial clusters and 1 other cluster, and the low IRScore group comprised 6 immune cell clusters, 18 epithelial clusters, 3 fibroblast clusters, and 4 endo_fibro clusters and 1 other cluster (**Figure S3G**). Consistently, the immune cell clusters had the highest IRScore, followed by epithelial clusters. Fibroblast and endo_fibro clusters presented the lowest IRScore (**Figures S3F, H**). Moreover, we calculated IRScore for each patient based on average gene expression and classified 62 patients into high and low IRScore groups. Patients with high IRScore had higher frequency of immune cells, while fibroblasts were enriched in low IRScore group (**Figure 3D**). We also evaluated gene expression between the two groups, and it showed that patients in the high IRScore group displayed significantly higher expression of immune checkpoint genes *CTLA4*, *HAVCR2*, *ICOS*, *LAG3*, *PDCD1* and *TIGIT*. Besides, expression of T cell development-associated genes *TBX21* and *EOMES*, tumor reactivity-associated genes *ITGAE* and *ENTPD1*, and cytotoxic gene *GZMB* were also significantly higher in patients with high IRScore (**Figure 3E**).

Collectively, we observed significant differences in transcriptomic, genomic and immune characteristics between the two groups. Mesenchymal and tumor-promoting phenotypes represented the low IRScore tumors, and in contrast, the high IRScore tumors displayed immune-active characteristics.

### CRC TILs’ clustering and subtype analysis

**Figure 4:**
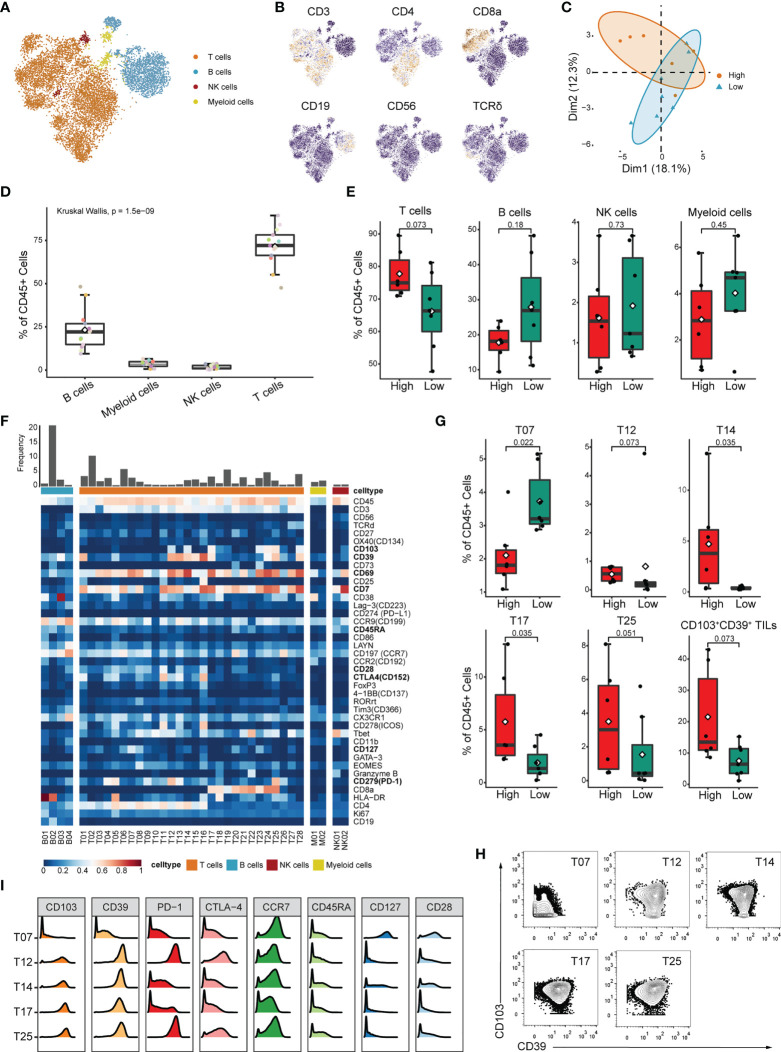
Identification of detailed immune profiles of CRC TILs by mass-cytometry. **(A)** tSNE plot displaying T cells, B cells, NK cells and myeloid cells based on manual annotation. **(B)** tSNE plots of normalized expression of markers used to annotate main immune clusters. **(C)** PCA analysis of cluster frequencies in the two IRScore groups. Each dot represented one patient. **(D)** Boxplot showing the frequency of B cell, myeloid cell, NK cell and T cell for each sample. **(E)** Comparison of frequencies of T cell, B cell, NK cell and myeloid cell between high and low IRScore groups. **(F)** Heatmap of normalized marker expression for 36 immune clusters. **(G)** Boxplots showing significant differences of frequency in T07, T12, T14, T17, T25 and total CD103^+^CD39^+^ TILs (T12+T14+T17+T24+T25) between high and low IRScore groups. **(H)** Contour plots showing expression of CD103 and CD39 in T07, T12, T14, T17 and T25. **(I)** Histograms showing expression of indicated markers in T07, T12, T14, T17 and T25.

Although high IRScore closely correlated with several immune signatures (**Figure 3C**), the detailed immune phenotype was still missing. We collected 13 treatment-naive CRC samples and performed bulk RNA-seq and CyTOF for TILs. We calculated IRScore for each patient according to RNA-seq data and divided them into high and low IRScore groups. Using the t-distributed stochastic neighbor embedding dimensionality reduction algorithm, we visualized the diversity of CD45^+^ tumor-infiltrating cells. We identified 4 major clusters: T cells, B cells, natural killer (NK) cells and myeloid cells (**Figures 4A, B**). PCA analysis of cell frequencies showed that patients in high and low IRScore groups were clearly separated (**Figure 4C**). T cells were the most abundant immune cell population among TILs, with a mean of 70% across samples, followed by B cells with a mean of 24% (**Figure 4D**). Besides, frequency of T cells was higher in the high IRScore group than that in the low IRScore group (*P*-value = 0.073). No significant difference was observed for frequencies of B cells, NK cells, or myeloid cells between the two groups (**Figure 4E**).

To further investigate functional subtypes of the overall TILs, an in-depth clustering analysis was conducted, and those TILs were finally classified into 36 clusters (**Figure 4F**). Specifically, we identified 4 B cell clusters (B01-B04), 2 myeloid cell clusters (M01-M02), 2 NK cell clusters (NK01-NK02), 16 CD4^+^ T cell clusters (T01-T16), 9 CD8^+^ T cell clusters(T17-T25), 2 γδ T cell clusters (T27-T28) and 1 CD4^-^CD8^-^ T cell cluster (T26). Most T cell clusters presented effector memory phenotype (Tem: CCR7^-^, CD45RA^-^) or central memory phenotype (Tcm: CCR7^+^, CD45RA^-^) (42) and expressed classical activation marker CD69 (43) (**Figure 4F**). We compared frequency of each cluster between high and low IRScore groups. Except for clusters M01, T07, T12, T14, T17 and T25, most clusters showed no significant difference between the two groups (**Figures 4G**, **S4A**). Intriguingly, clusters with higher frequencies in the high IRScore group (T12, T14, T17 and T25) were positive for CD103 and CD39 (**Figure 4H**). Frequencies of total CD103^+^CD39^+^ TILs were also significantly higher in the high IRScore group (**Figure 4G**, *P*-value = 0.073). Previous studies revealed that co-expression of CD103 and CD39 identified a unique population of tumor-reactive CD8^+^ TILs in solid human tumors with an exhausted tissue-resident memory phenotype (44, 45). T25 exhibited high expression of exhaustion markers PD-1, CTLA4 and low expression of CCR7, CD45RA, CD127 and CD28, representative of an effector-memory phenotype (**Figure 4I**). T12 was the CD4^+^ counterpart of T25, with an almost identical marker expression pattern (**Figures 4F, I**). CD39^+^CD4^+^ TILs shared similar activated, tissue-resident and effector cell-associated signatures with CD39^+^CD103^+^CD8^+^ TILs (46). The CD4^+^ T14 and CD8^+^ T17 were also inter-counterparts (**Figures 4F, I**). Together, clusters co-expressing CD103 and CD39 had higher frequencies in the high IRScore group. This phenotype might be relevant to better prognosis in CRC patients with higher IRScore.

### The characteristics of CD103^+^CD39^+^CD4^+^/CD8^+^ T cells and relationship with IRScore

To further evaluate whether CD103^+^CD39^+^ T cells represented the immune signature of the high IRScore group, we calculated IRScore of tumor-infiltrating CD4^+^/CD8^+^ T cells from a single-cell dataset (GSE108989) and investigated gene expression patterns of CD4^+^/CD8^+^ TILs co-expressing CD103 (*ITGAE*) and CD39 (*ENTPD1*). We classified those TILs into high and low IRScore groups. Besides, we defined cells expressing CD103 and CD39 as double-positive cells (DP), cells expressing neither CD103 nor CD39 as double-negative cells (DN), and cells expressing either CD103 or CD39 as single-positive cells (SP).

**Figure 5:**
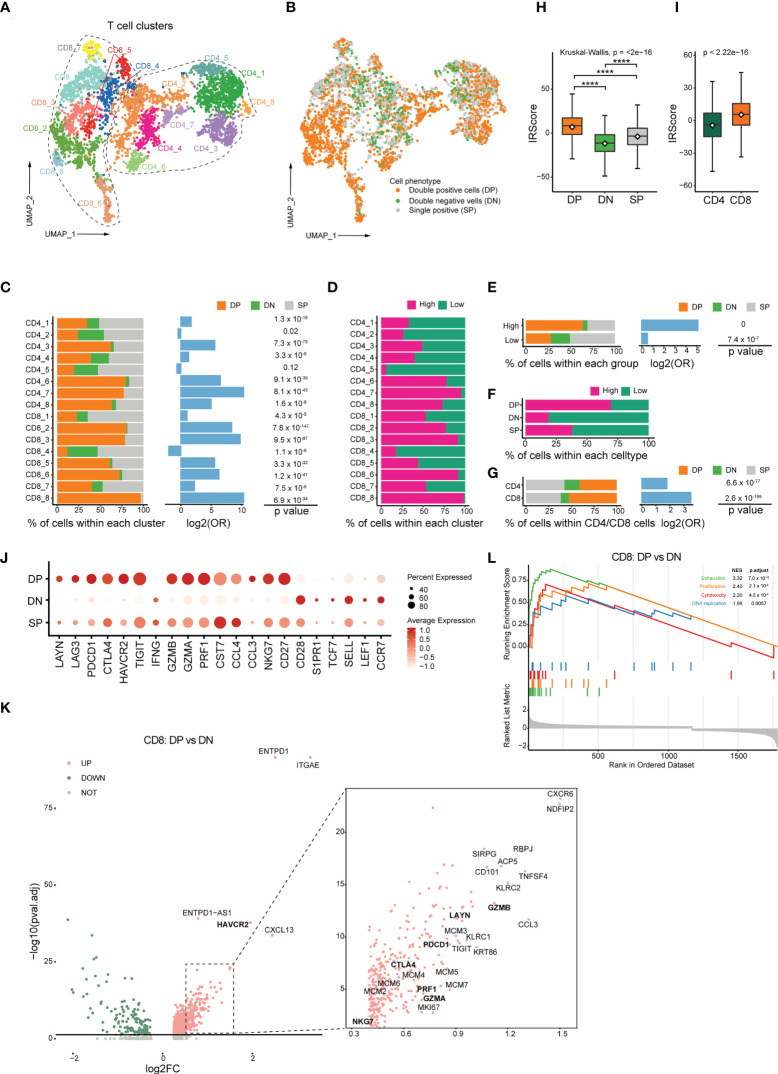
Transcriptomic profiles of CD103^+^CD39^+^ T cells and association with IRScore. **(A)** UMAP projection of CD4^+^ and CD8^+^ TILs, showing 8 CD8^+^ clusters and 8 CD4^+^ clusters in different colors. **(B)** UMAP projection of CD103 and CD39 double-positive cells (DP), double-negative cells (DN) and single-positive cells (SP). **(C)** Stacked barplot showing the percentile of DP, DN and SP cells in each clusters (left), log2 odds ratio (DP versus DN, middle) and p value (right). **(D)** Percentile of high and low IRScore TILs in each cluster. **(E)** Stacked barplot showing the percentile of DP, DN and SP cells in high and low IRScore groups (left), log2 odds ratio (DP versus DN, middle) and p value (right). **(F)** Percentile of high and low IRScore TILs among three cell types. **(G)** Stacked barplot showing the percentile of DP, DN and SP cells in CD4^+^ and CD8^+^ TILs (left), log2 odds ratio (DP versus DN, middle) and p value (right). **(H)** Comparison of IRScore among DP, DN and SP cells. **(I)** Comparison of IRScore between CD4^+^ and CD8^+^ TILs. **(J)** Dotplot showing expression of exhausted, cytotoxic and naive markers in DP, DN and SP cells. **(K)** Volcano plot of differentially expressed genes between CD8^+^ DP and DN cells. **(L)** Enrichment plot for the curated gene signatures in CD8^+^ TILs by GSEA. ****P < 0.0001

Sixteen clusters were identified, including 8 CD4^+^ clusters and 8 CD8^+^ clusters (**Figures 5A**, **S5A**). CD8^+^ clusters were identified as activated effector cytotoxic cells based on the expression of canonical cytotoxic markers *GZMA/B/H*, *PRF1*, *IFNG* and *NKG7*. CD8_2, CD8_6, and CD8_8 also exhibited expression of exhaustion markers *PDCD1* and *HAVCR2*, indicating dual characteristics. Among CD4^+^ clusters, CD4_2 and CD4_4 specifically expressed naive marker genes such as *CCR7*, *IL7R* and *SELL*, thus representing naive T cells; CD4_1, CD4_3, CD4_5 and CD4_8 were characterized by high expression of *FOXP3* and *IL2RA*, suggestive of the identity of regulatory T cells (Tregs); CD4_6 and CD4_7 were comprised of CD4^+^ T cells with high expression of exhausted marker genes *HAVCR2*, *PDCD1* and cytotoxic molecules *GZMA*, *GZMB*, *PRF1*, indicative of the status of exhausted and cytotoxic CD4^+^ T cells (**Figure S5B**).

DP cells comprised 46.2% of total detected cells and were significantly enriched in the high IRScore group with absolute predominance over DN cells. In contrast, DN cells only constituted 13.3% of whole cells. There were relatively equal proportion of DP and DN cells in the low IRScore group (**Figures 5B, E**). Besides, DP cells were enriched in 6 CD4^+^ and 7 CD8^+^ clusters (**Figure 5C**). Surprisingly, a roughly coincident ratio could be observed when comparing the distribution of high and low IRScore TILs in each cluster (**Figure 5D**). Moreover, DP cells were composed of the most significant proportion of high IRScore cells, and DN cells were the least, consistent with the observation that DP cells displayed the highest IRScore, with DN cells being the lowest, and SP cells having intermediate IRScore (**Figures 5F, H**). We also compared IRScore between CD4^+^ and CD8^+^ cells and found that CD8^+^ cells had significantly higher IRScore than CD4^+^ cells, on account of a higher percentage of DP cells among CD8^+^ cells (**Figures 5G, I**).

We examined the expression of well-known naive, cytotoxic and exhausted markers among DP, DN and SP cells (45). Intriguingly, DP cells displayed dual phenotypes with higher expression of cytotoxic and exhausted marker genes, whereas DN cells had higher expression of naive genes; SP cells exhibited an intermediate status (**Figure 5J**). To better understand the function of DP cells, we compared them with DN cells at the transcriptome level. Among CD8^+^ T cells, DP cells highly expressed a set of 898 genes (adjusted *P*-value< 0.05, log2FC > 0), including exhausted markers (*HAVCR2*, *LAYN*, *TIGIT*, *PDCD1*, *CTLA4*), cytotoxic markers (*GZMA*, *GZMB*, *PRF1*, *NKG7*) and proliferation-related genes MCMs (**Figure 5K**). The concurrence of an exhausted phenotype with cytotoxic and proliferative characteristics in DP cells was further confirmed by GSEA analysis of HALLMARK gene sets and the curated signatures. Genes up-expressed among DP cells were significantly enriched for processes associated with inflammation, proliferation, cytotoxicity and presented an exhausted phenotype (**Figures 5L**, **S5E**). CD4^+^ DP cells also exhibited an exhausted and proliferative phenotype (**Figures S5C, D**). GSEA analysis of HALLMARK gene sets and the curated gene signatures revealed that immune-related pathways were enriched considerably in up-expressed genes in CD4^+^ DP cells, and an unfavorable status of hypoxia and unfolded protein response was likewise found in CD4^+^ DN cells (**Figure S5F**). Together, these data indicated that DP cells were accumulated in the high IRScore group, characteristic of a cytotoxic, exhausted and proliferative phenotype.

### CD45^+^CD3^+^CD103^+^CD39^+^ signature could predict the CRC prognosis and responses to ICB therapy

**Figure 6:**
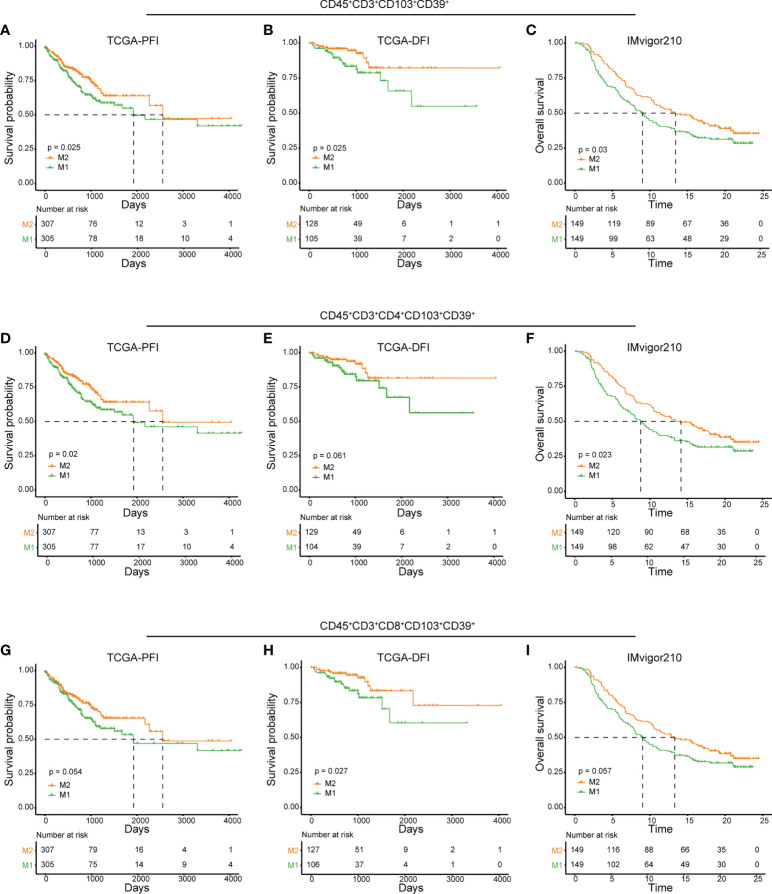
Predictive value of CD45^+^CD3^+^CD103^+^CD39^+^ phenotype in prognosis and immunotherapy. **(A, D, G)** Kaplan-Meier plots of relationship between the immune signatures from RNAseq dataset (CD45^+^CD3^+^CD103^+^CD39^+^ **(A)**, CD45^+^CD3^+^CD4^+^CD103^+^CD39^+^ **(D)** and CD45^+^CD3^+^CD8^+^CD103^+^CD39^+^ **(G)** and PFI in TCGA cohort. Patients were stratified according to the median of signature NES, where M1 was the lower NES and M2 was the higher NES. B, E, **(H)** Kaplan-Meier plots of relationship between the immune signatures from RNAseq dataset (CD45^+^CD3^+^CD103^+^CD39^+^ **(B)**, CD45^+^CD3^+^CD4^+^CD103^+^CD39^+^ **(E)** and CD45^+^CD3^+^CD8^+^CD103^+^CD39^+^ **(H)** and DFI in TCGA cohort. Patients were stratified according to the median of signature NES, where M1 was the lower NES and M2 was the higher NES. C, F, **(I)** Kaplan-Meier plots of relationship between immune signatures from RNAseq dataset (CD45^+^CD3^+^CD103^+^CD39^+^ **(C)**, CD45^+^CD3^+^CD4^+^CD103^+^CD39^+^ **(F)** and CD45^+^CD3^+^CD8^+^CD103^+^CD39^+^ **(I)** and OS in IMvigor210 cohort. Patients were stratified according to the median of signature NES, where M1 was the lower NES and M2 was the higher NES.

As identified in CyTOF and scRNA-seq analyses, high IRScore could be partially represented by a CD45^+^CD3^+^CD103^+^CD39^+^ signature, we further examined its predictive value and efficacy in predicting response to ICB therapy. Similarly, we calculated NES of this CD45^+^CD3^+^CD103^+^CD39^+^ signature and divided patients into two groups (M1 and M2) according to the median value. In TCGA cohort, patients with higher NES presented prolonged survival than those with lower NES (**Figures 6A, B**). In ICB therapy cohort IMvigor210, patients with higher NES had prolonged overall survival, and NES of patients with better responses (CR/PR/SD) to ICB therapy were significantly higher than those with progressive disease (PD). Besides, the higher NES group (M2) consisted of more patients that benefited from ICB therapy (**Figures 6C**, **S6A-C**). We also showed that CD103^+^CD39^+^ CD4^+^/CD8^+^ T cells had certain ability in predicting prognosis and responses to ICB therapy respectively (**Figures 6D-I**, **S6D-I**). Thus, the CD45^+^CD3^+^CD103^+^CD39^+^ signature was a beneficial immune signature that, to a certain extent, could predict the prognosis and efficacy of ICB therapy.

## Discussion

The TME is a heterogeneous mixture of tumor cells, infiltrating and resident host cells, extracellular matrix and secreted cytokines (9–12). Cross-talk between TME components significantly affects tumor development and progression (47). This study utilized immune-related signatures to develop IRScore for CRC patients’ stratification. Investigations regarding the relationship between this classification strategy and prognosis, transcriptomic profiles, immune activities etc., will allow for a better understanding of the TME and improved instructions for ICB therapy (**Figure S1A**).

In this study, we developed the IRScore system based on 54 prognosis-related immune signatures. Our research revealed that IRScore was positively correlated with immune and proliferative signatures and negatively associated with EMT and naiveness (**Figure 1E**). The proliferation signature of high IRScore might be a result of CD8^+^ TILs, however, alternative interpretation is the proliferating tumor cells. Actively proliferating cells are more likely to be accumulated with mutations, leading to a heavier mutation burden. T cells’ recognition of mutation-derived neoantigens in tumors is critical for antitumor activity. Moreover, tumor neoantigens are associated with therapeutic benefits in PD-1 or CTLA-4 blockade. Besides, IRScore was positively correlated with prolonged survival and beneficial responses to ICB therapy, which might be explained by favorable transcriptomic features and active antitumor activities in the high IRScore group. Better responses to ICB treatment in the high IRScore group allowed us to focus on the differences of infiltrating immune signatures in TME between the two groups.

As indispensable components to the TME, TILs show a critical role in tumor progression. The antitumor function involves CD8^+^ cytotoxic cells, NK cells, and CD4^+^ Th1 cells, while MDSCs, TAMs and Tregs inhibit antitumor responses (48–51). We showed that the high IRScore group was more abundant for several CD8^+^ T cell populations and Th1 cells, whereas the low IRScore group had more fibroblasts, endothelial cells and pericytes (**Figure 3C**). We found more Tregs in the high IRSore group, which might be due to Tregs displaying positive effects and associating with good prognosis in specific cancer types (52–54). Analyses of a CRC single-cell dataset confirmed the fact that endothelial and fibroblast clusters had the lowest IRScore while immune cell clusters were the highest (**Figures 3C, E**).

Recently, molecular subtyping systems focusing on immune infiltrating signatures have emerged to stratify patients and predict prognosis and immunotherapeutic outcomes in various tumors (55–57). However, these subtyping systems failed to depict detailed immune profiles in TME, which are important for understanding the mechanisms by which immune cells modulate tumor progression and response to ICB therapy. To solve this problem, we used mass cytometry to perform in-depth immune profiling of tumor samples from CRC patients. Among the 36 identified clusters, T12, T14, T17 and T25 were significantly abundant in the high IRScore group, and all of them displayed a CD103^+^CD39^+^ phenotype (**Figures 4F-H**). Since high IRScore was associated with favorable prognosis and antitumor response, these clusters might represent tumor-reactive populations. T25 exhibited increased expression of PD-1, CTLA4 and low expression of CCR7, CD45RA, CD127 and CD28 (**Figure 4I**), consistent with previous studies that a subset of tumor-reactive CD8^+^ TILs were positive for CD103 and CD39 and exhibited an exhausted tissue-resident memory phenotype (44, 45). We further validated CD4^+^ and CD8^+^ TILs co-expressing CD103 and CD39 at the single-cell level. Duhen and colleagues found that CD103^+^CD39^+^CD8^+^ TILs were enriched in CRCs with MSI/dMMR and displayed more elevated exhaustion markers *CTLA4, PDCD1, HAVCR2* and lower expression of naive markers *S1PR1, SELL, TCF7* (44). We showed that CD8^+^ DP TILs up-regulated genes related to proliferation, exhaustion, cytotoxicity markers and down-regulated genes related to naiveness (**Figures 5J-L**). Moreover, CD103^+^CD39^+^CD8^+^ TILs displayed more clonal expansion and better tumor antigen recognition than CD103^-^CD39^-^CD8^+^ TILs, indicating that CD103^+^CD39^+^CD8^+^ TILs were enriched with tumor specific T cells (44, 58). *CXCL13*, a B-cell recruiting chemokine, was the most significantly up-regulated gene except *HAVCR2*, *ITGAE* and *ENTPD1* in CD8^+^ DP cells (**Figure 5K**). He et al. showed that CXCL13 was the unique marker for tumor antigen specific CD4^+^/CD8^+^ T cells in multiple tumors (59). Besides, CXCL13^+^CD103^+^CD8^+^ TILs were potentially associated with B cell recruitment, neoantigen load and tertiary lymphoid structures (TLSs) formation in human tumors (60), and were identified as tumor antigen specific T cells in lung cancer (59, 61). Thus, CD103 and CD39 positive cells might shape a tumor microenvironment suitable for B-cell antitumor activities.

Other researchers had shown the effects of CD103^+^CD39^+^ TILs in various tumor types. This population of T cells were enriched in genes associated with exhaustion, and may represent a prognostic marker of cancer progression (44, 58). Higher frequencies of CD39^+^CD103^+^CD8^+^ TILs in patients with head and neck cancer were associated with better overall survival, and *in vitro* studies showed that co-expression of CD39 and CD103 were strongly enriched in tumor-recognizing and -killing CD8 T cells (44). Circulating CD103^+^CD39^+^CD8^+^ T cells was significantly enriched in nasopharyngeal carcinoma patients without distant metastasis, and those patients had better PFS (62). CD103^+^CD39^+^ TILs could also be considered as a potential biomarker for predicting patients’ response to novel ICB approaches in various tumors. CD103^+^CD39^+^ TILs could serve as a potential biomarker of anti-OX40 clinical activity in patients with head and neck cancer, and might represent a biomarker of RFS following anti-PD-1 therapy in melanoma (63, 64). In our study, we also revealed that the CD45^+^CD3^+^CD103^+^CD39^+^ signature could predict prognosis and response to ICB (**Figure 6**). These findings suggest that co-expression of CD39 and CD103 could serve as useful markers of tumor specific CD8^+^ T cells, and could be exploited for the development of targeted immunotherapies.

However, due to the limitation of CyTOF samples and markers, we only observed limited immune features that were significantly differentiated between high and low groups. Other potential phenotypes might have a significant difference if more samples and markers were included. Besides, more in-depth investigations and basic experimental research are required to explain further the underlying mechanisms of the event in the future.

In conclusion, we developed IRScore to stratify CRC patients and explored various profiles contributing to the differences between high and low IRScore groups. We further characterized the detailed immune signature, CD103^+^CD39^+^ T cells, in IRScore system, which may offer important clues to mechanisms of antitumor immune responses in CRC.

## Data availability statement

The datasets presented in this study can be found in online repositories. The names of the repository/repositories and accession number(s) can be found in the article/**Supplementary Material**. The RNA-seq data of 13 CRC patients presented in the study are deposited in NCBI Sequence Read Archive, accession number PRJNA884190.

## Ethics statement

The studies involving human participants were reviewed and approved by Institutional Review Board of the First Affiliated Hospital of Zhejiang University. The patients/participants provided their written informed consent to participate in this study.

## Author contributions

XZ, DN, and CL led and supervised the study as joint authors. YL performed public datasets collection, bioinformatic analyses and generated the figures and tables; YZ performed CyTOF experiments; HH collected tumor samples; MG and QP assisted the experiments. YL wrote the manuscript and XZ, DN, and CL revised the manuscript. All authors contributed to the article and approved the submitted version.

## Funding

This work was supported by National Natural Science Foundation of China (31870899, 32070899 to XZ; 82272126 and 31301100 to CL; 82103304 to QP), CAMS Innovation Fund for Medical Sciences (2019-I2M-5-045) to XZ and the Independent Task of State Key Laboratory for Diagnosis and Treatment of Infectious Diseases (2022zz07 to QP).

## Acknowledgments

We would like to thank all staff members and website maintainers of the TCGA program, GEO data repository and UCSC Xena data portal, and all authors who made their research work public. We thank Dr. Kangxin He for collecting clinical samples. We thank the technical support for CyTOF by Zhejiang Puluoting Health Technology Co., Ltd (Hangzhou, Zhejiang, China). We thank the technical support by the Core Facilities, Zhejiang University School of Medicine. We thank Dr. Hangjun Wu in the Center of Cryo-Electron Microscopy (CCEM), Zhejiang University for his technical assistance on computer clustering. CL is supported by Alibaba Cloud.

## Conflict of interest

The authors declare that the research was conducted in the absence of any commercial or financial relationships that could be construed as a potential conflict of interest.

## Publisher’s note

All claims expressed in this article are solely those of the authors and do not necessarily represent those of their affiliated organizations, or those of the publisher, the editors and the reviewers. Any product that may be evaluated in this article, or claim that may be made by its manufacturer, is not guaranteed or endorsed by the publisher.

## Supplementary material

Supplementary Figure 1 Association between IRScore and clinical factors and molecular signatures. (**A)**. Workflow of this study: IRScore construction and characterization, and IRScore-based detailed immune signatures exploration. **(B)**. Kaplan-Meier curves of recurrence-free survival based on IRScore groups in 7 GEO cohorts. **(C)**. Kaplan-Meier curves of recurrence-free survival based on IRScore groups in a combined dataset. **(D, E)**. Kaplan-Meier curves of disease-specific survival (DSS, **D**) and overall survival (OS, **E**) based on IRScore groups in TCGA cohort. **(F)**. Violin plots showing the relationship between IRScore and dMMR/pMMR (left), tumor stages (middle) and Sankey diagram (right) illustrating the relationship between IRScore and CRC subtypes in GSE39582 cohort. **(G)**. The association and correlation between IRScore and different gene signatures (Pan-F-TBRS, naiveness, plasminogen inhibitor, CD8 T effector, cell cycle, cytotoxicity, DNA damage repair, DNA replication, homologous recombination, immune checkpoint, mismatch repair, nucleotide excision repair and exhaustion) in TCGA cohort.

Supplementary Figure 2 Prediction of response to immunotherapy using SubMap. (**A, B)**. SubMap prediction of response to anti-PD1 and anti-CTLA4 treatment in TCGA cohort **(A)** and GSE39582 cohort **(B)**.

Supplementary Figure 3 Transcriptomic, genomic and immune characterizations of IRScore. (A). Circos plots of GSEA-based Cancer Hallmarks pathways (top) and KEGG pathways (bottom) differences between high and low IRScore groups (from the innermost to the outmost: TCGA, GSE39582, GSE39084, GSE38832, GSE37892, GSE33113, GSE17538, GSE103479). The grids were colored according to NES (high versus low IRScore group, p.adj< 0.1). A blank grid meaning the pathway difference is not significant (p.adj > 0.1). **(B)**. Volcano plot showing up- or down-regulated genes of high versus low IRSore group in TCGA cohort (p.adj< 0.05, absolute log2FoldChange > 1). Corresponding bar plots showing the enriched KEGG pathways in up- or down-regulated genes. **(C)**. Boxplot showing the difference of mutation burden between high and low IRScore groups in TCGA cohort. **(D)**. Dotplot showing the expression of genes used to identify endothelial cells, epithelial cells, fibroblasts and immune cells. **(E)**. The tSNE projection of endo-fibro cells, epithelial cells, fibroblasts and immune cells. **(F)**. The tSNE projection of cluster-level IRScore. **(G)**. Stacked bar chart showing the cluster compositions in high and low IRScore groups. **(H)**. Comparison of IRScore among main cell types. Each point represented one cell subset.

Supplementary Figure 4 Difference of cluster frequencies between high and low IEScore groups. (A). Boxplots showing the difference of frequencies in 31 immune clusters between high and low IRScore groups.

Supplementary Figure 5 Transcriptomic profiles of CD103^+^CD39^+^ T cells. (A). Violin plot showing the expression of CD69, CD4, CD8A and CD8B. **(B)**. Heatmap of CD4^+^ and CD8^+^ T cells, with 16 main clusters identified, each containing a unique set of signature genes. **(C)**. Volcano plot of differentially expressed genes between CD4^+^ DP and DN cells. **(D)**. Enrichment plot for the curated gene signature in CD4^+^ TILs by GSEA. **(E, F)**. Gene set enrichment analysis of Cancer Hallmark gene sets identified significantly enriched pathways in CD8^+^ **(E)** and CD4^+^ **(F)** DP and DN cells.

Supplementary Figure 6 Prediction of CD45^+^CD3^+^CD103^+^CD39^+^ phenotype to immunotherapy. (A, D, G). Violin plots showing the difference of NES (CD45^+^CD3^+^CD103^+^CD39^+^ **(A)**, CD45^+^CD3^+^CD4^+^CD103^+^CD39^+^ **(D)** and CD45^+^CD3^+^CD8^+^CD103^+^CD39^+^ **(G)**) between CR/PR/SD and PD in IMvigor210 cohort. **(B, E, H)**. Composition of patients’ responses to PD-L1 inhibitor treatment between M1 and M2 in IMvigor210 cohort (**(B)**: *P*-value = 0.010; **(E)**: *P*-value = 0.019; **(H)**: *P*-value = 0.035, Fisher’s exact test). **(C, F, I)**. Waterfall plots illustrating NES (CD45^+^CD3^+^CD103^+^CD39^+^ **(C)**, CD45^+^CD3^+^CD4^+^CD103^+^CD39^+^ **(F)** and CD45^+^CD3^+^CD8^+^CD103^+^CD39^+^ **(I)**) according to immunotherapeutic responses in IMvigor210 cohort.

## Glossary

**Table:**

CIMP	CpG island methylator phenotype
CIN	Chromosomal instability
CR	Complete response
CRC	Colorectal cancer
CTLA4	Cytotoxic T-lymphocyte associated protein 4
CTLs	Cytotoxic T lymphocytes
CyTOF	Cytometry by time of flight
DEGs	Differentially expressed genes
dMMR	Deficient mismatch repair
DN	Double-negative cells
DP	Double-positive cells
ECM	Extracellular matrix
EMT	Epithelial-mesenchymal transition
GEO	Gene Expression Omnibus
HBSS	Hank’s Balanced Salt Solution
HGSC	High-grade serous ovarian cancer
HR	Hazard ratio
ICB	Immune checkpoint blockade
IRScore	Immune-related signature score
MCM	Minichromosome maintenance
MSCs	Mesenchymal stem cells
MSI	Microsatellite instability
MSS	Microsatellite stability
MSigDB	Molecular Signatures Database
NES	Normalized enrichment score
NK	Natural killer cell
pan-F-TBRS	Pan-fibroblast TGF-β response signature
PD	Progressive disease
PD-1	Programmed cell death 1
pDCs	Plasmacytoid dendritic cells
PR	Partial response
pMMR	Proficient mismatch repair
RFS	Recurrence-free survival
scRNA-seq	Single-cell RNA sequencing
SD	Stable disease
SP	Single-positive cells
ssGSEA	Single sample gene set enrichment analysis
SubMap	Subclass mapping
Tcm	Central memory T cell
Tem	Effector memory T cell
Th1	Type 1 T helper cell
Th2	Type 2 T helper cell
TILs	Tumor infiltrating lymphocytes
TME	Tumor microenvironment
Tregs	Regulatory T cells
UPR	Unfolded protein response
