# Supplementary figures and images for: Immune-infiltrating signature-based classification reveals CD103+CD39+ T cells associate with colorectal cancer prognosis and response to immunotherapy
Source: Front Immunol. 2022 Oct 12;13:1011590. doi: 10.3389/fimmu.2022.1011590 (PMC9596778; doi:10.3389/fimmu.2022.1011590)

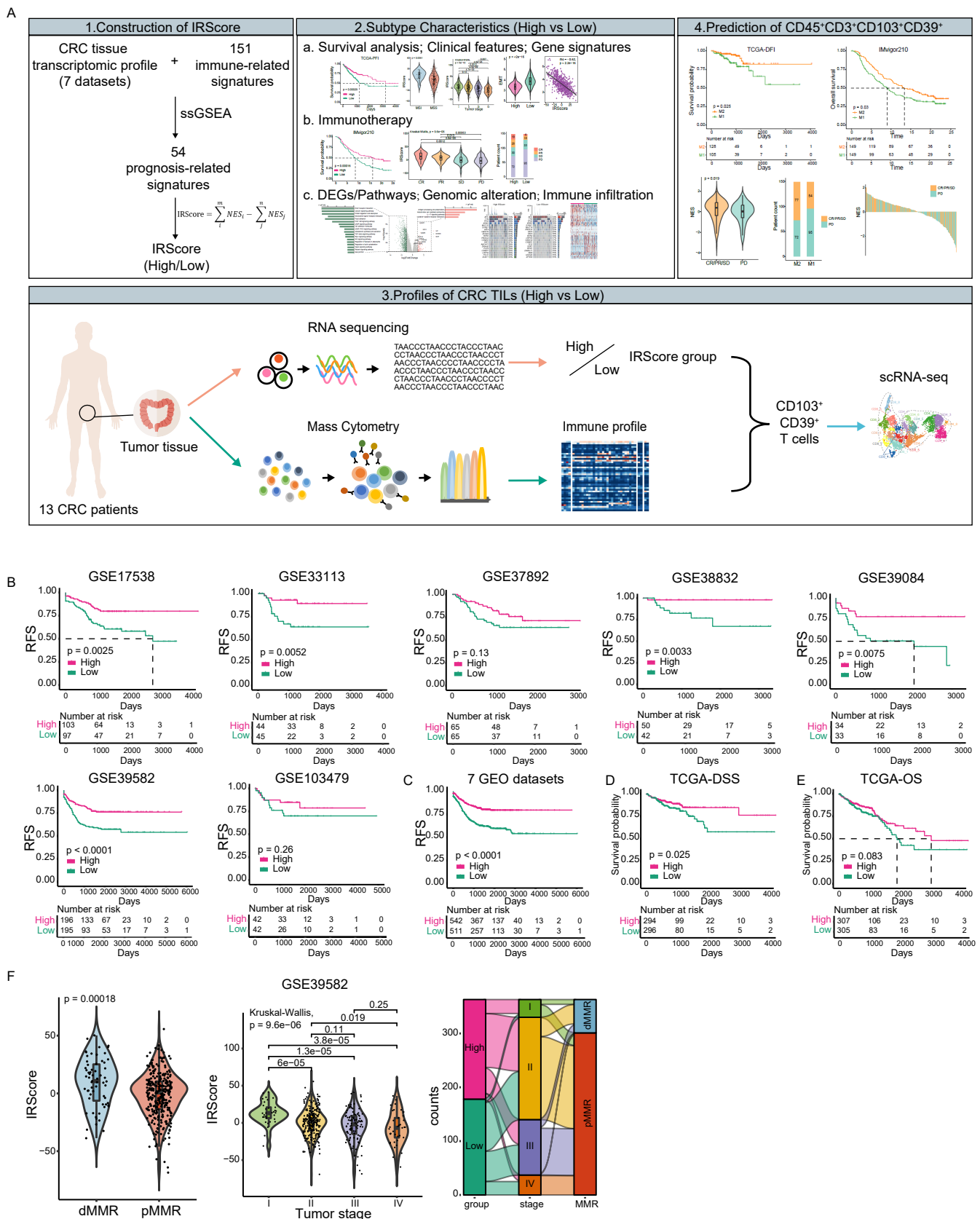

Figure-S1

G

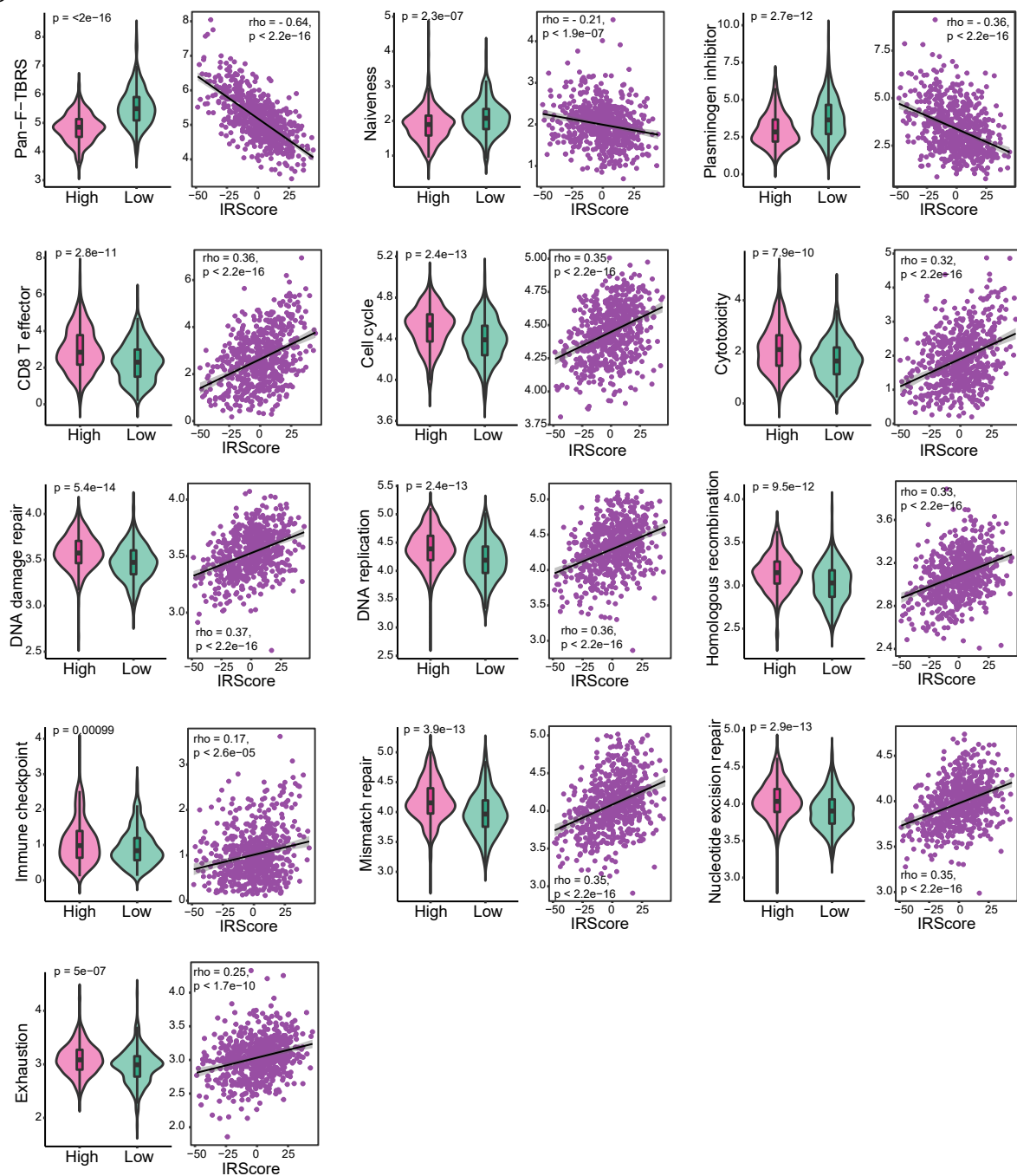

Figure-S1

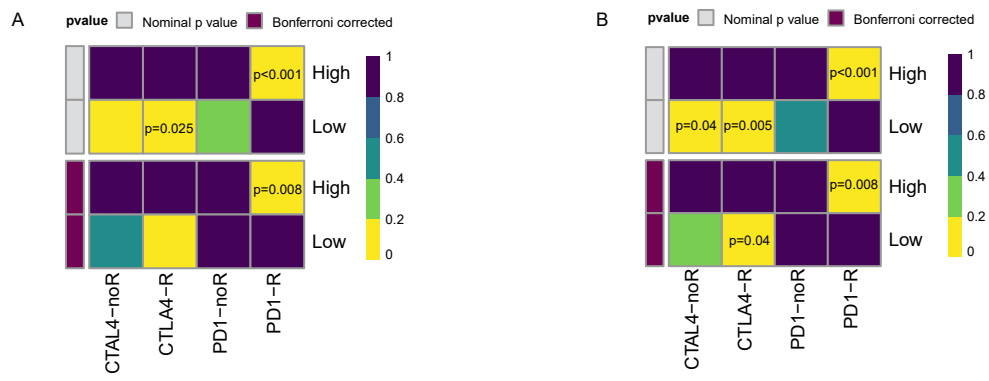

Figure-S2

A

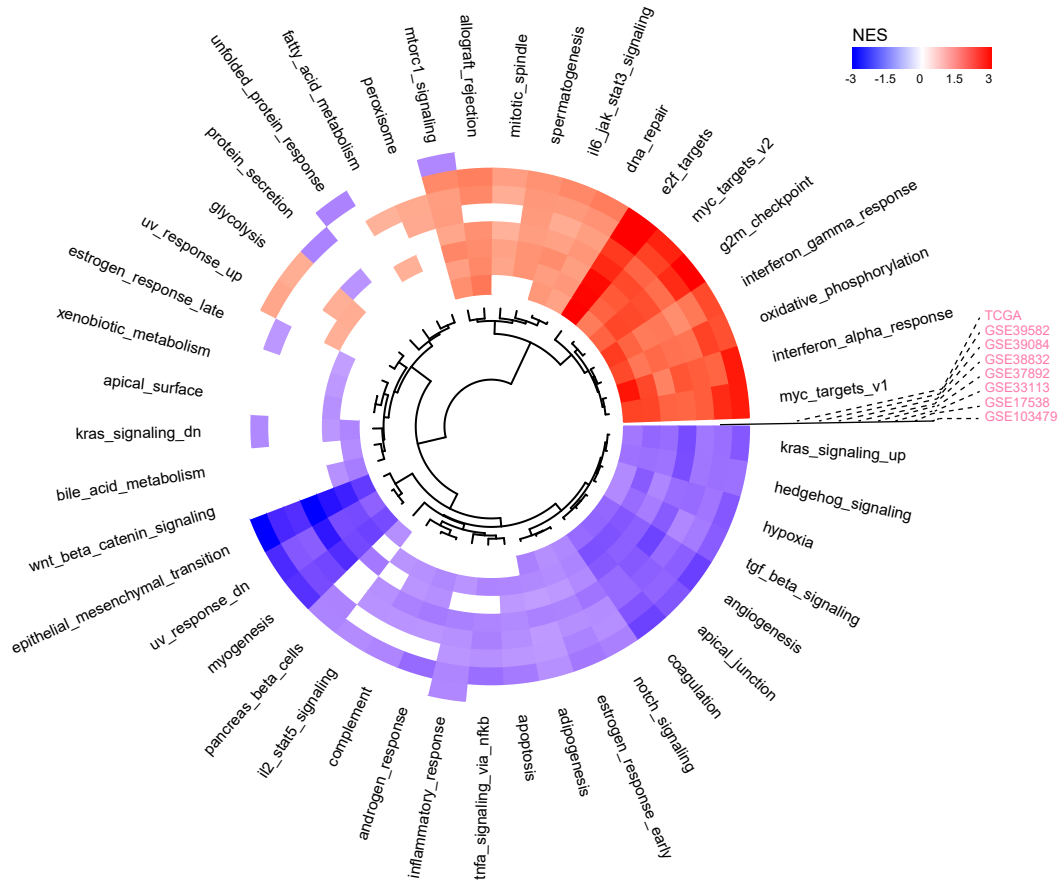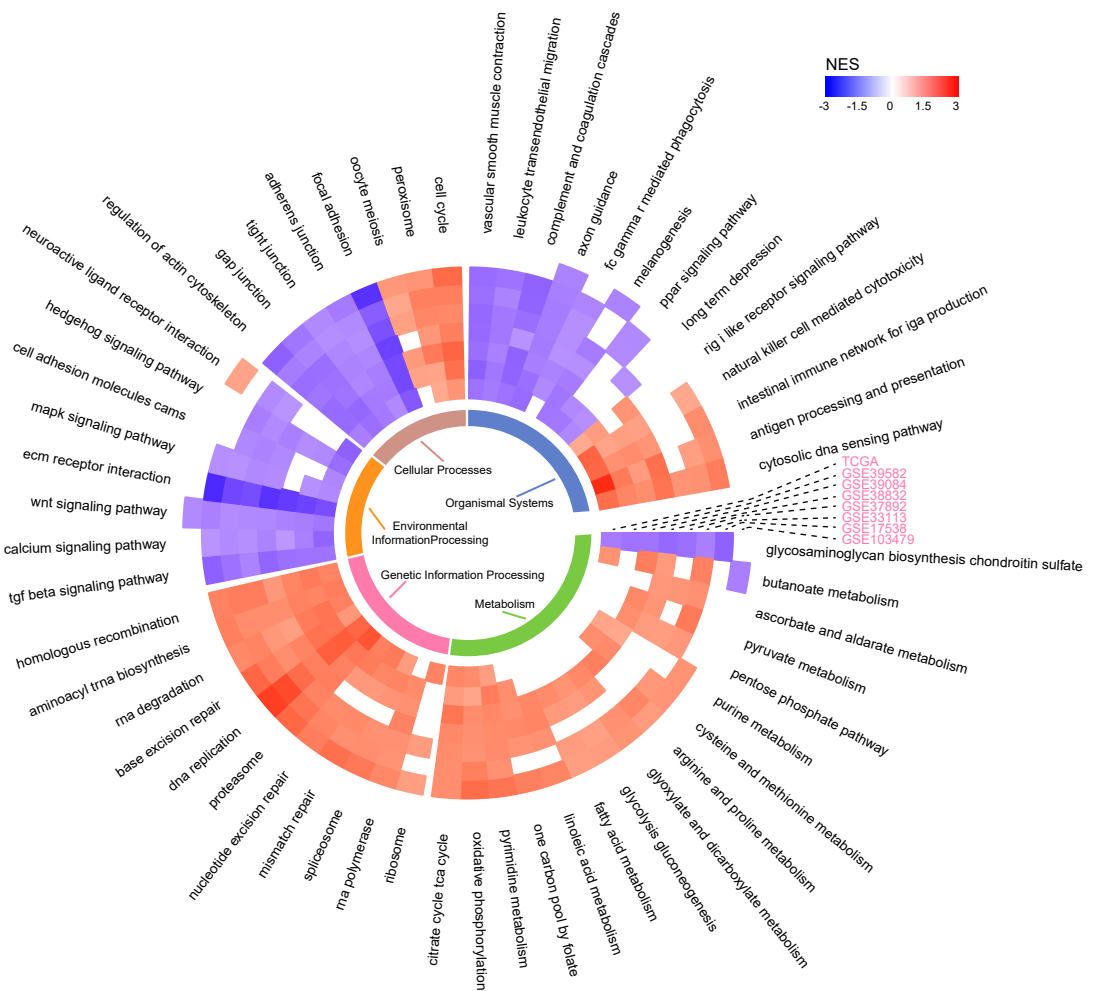

Figure-S3

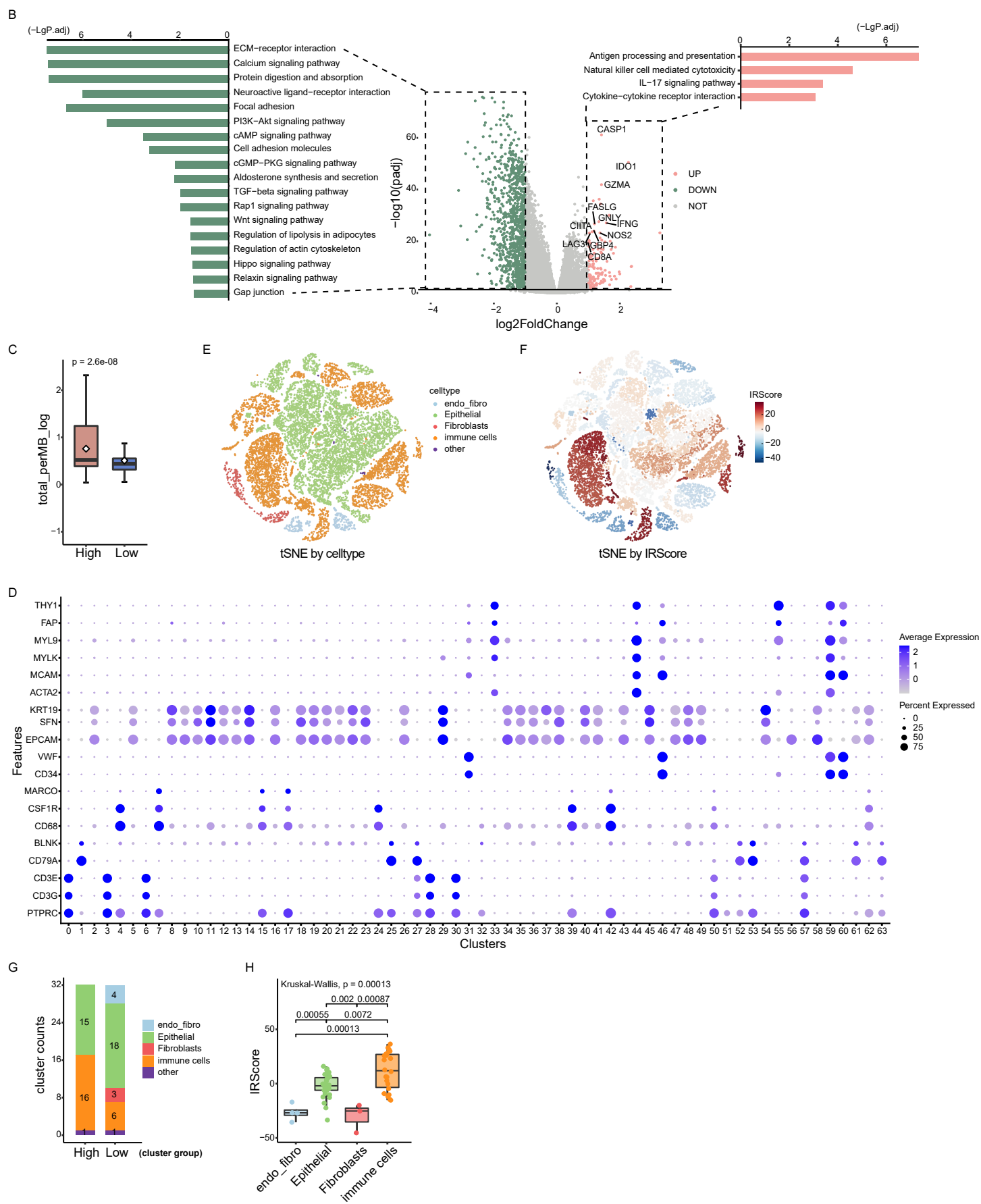

Figure-S3

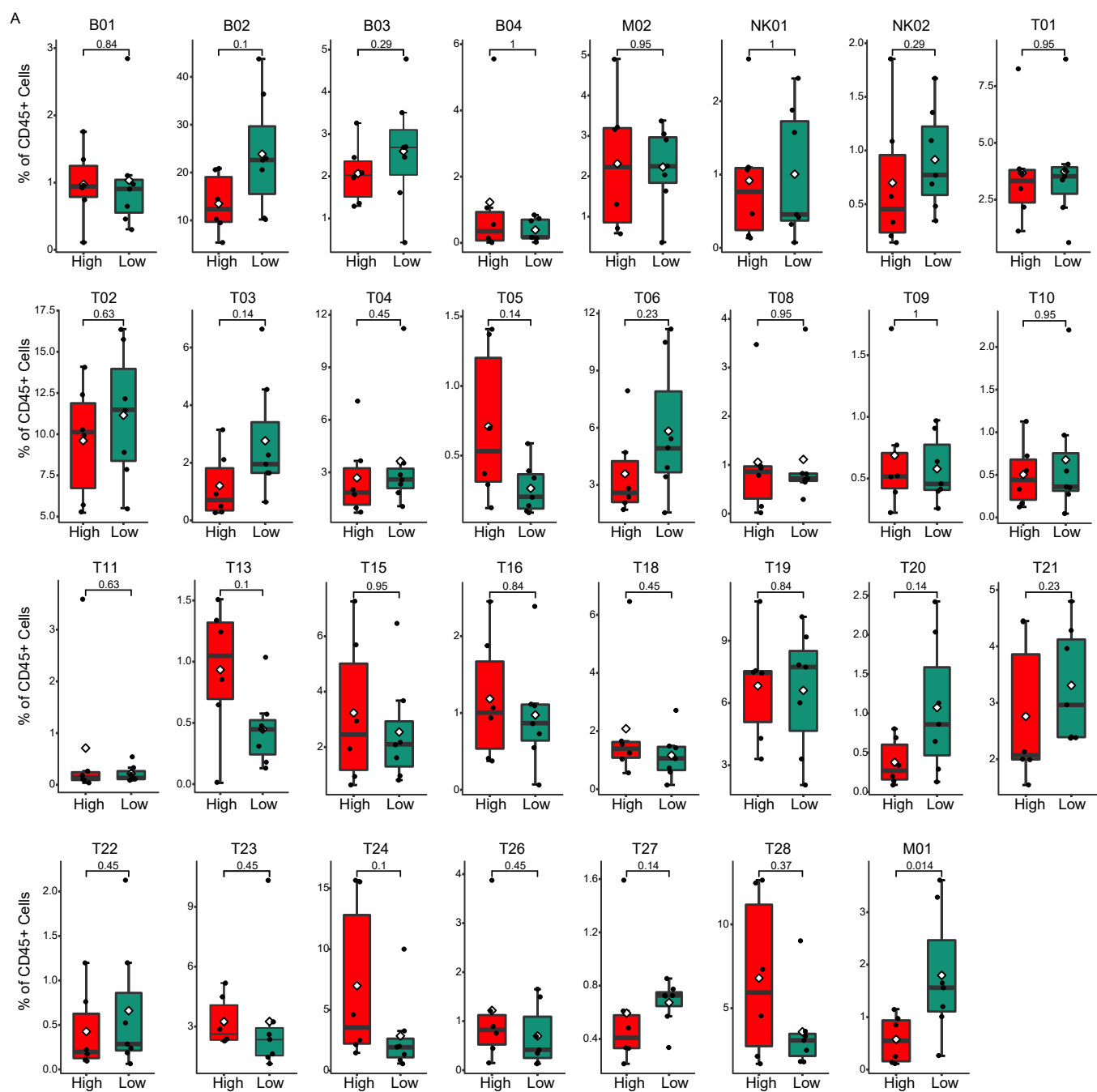

Figure-S4

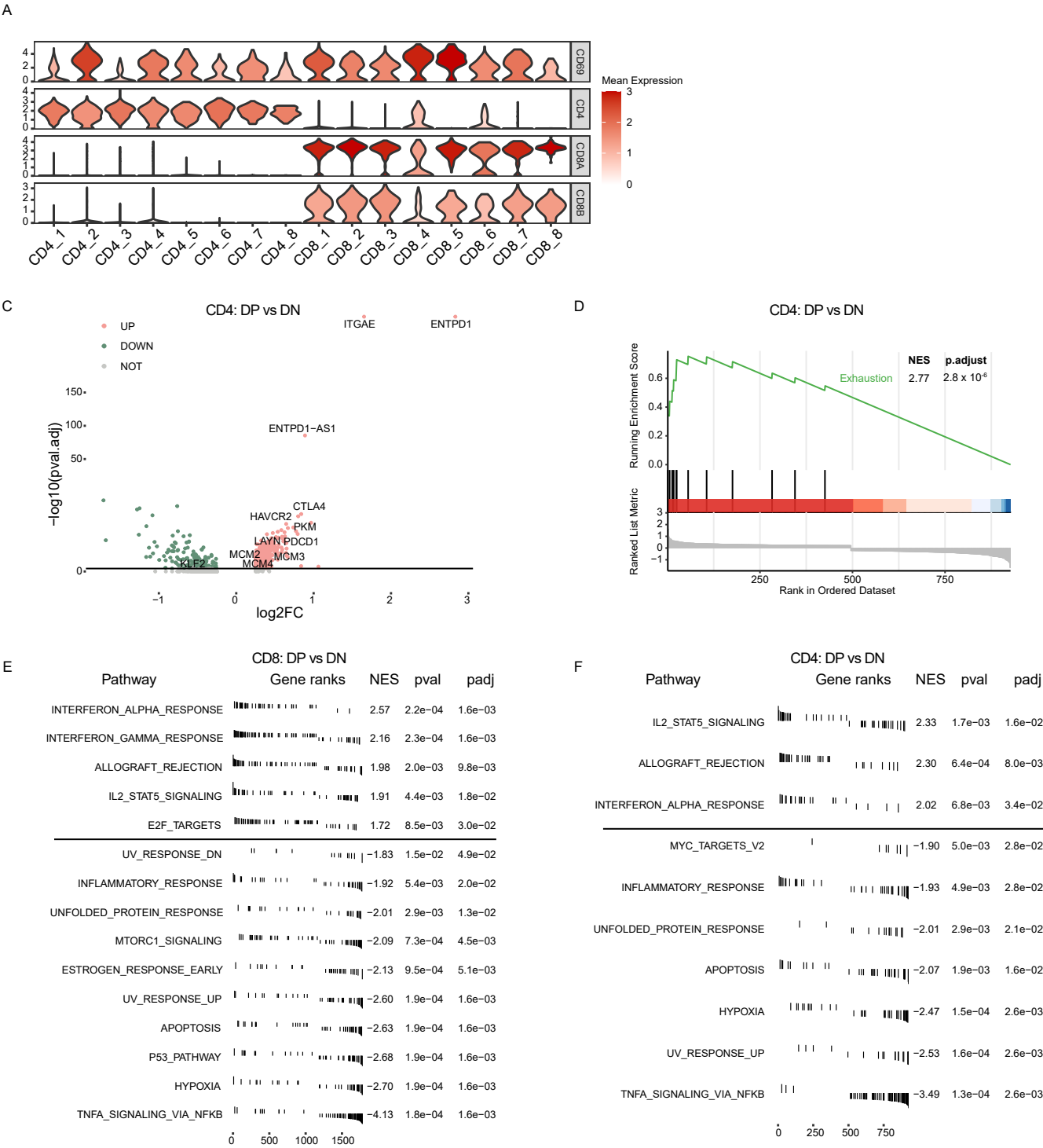

Figure-S5

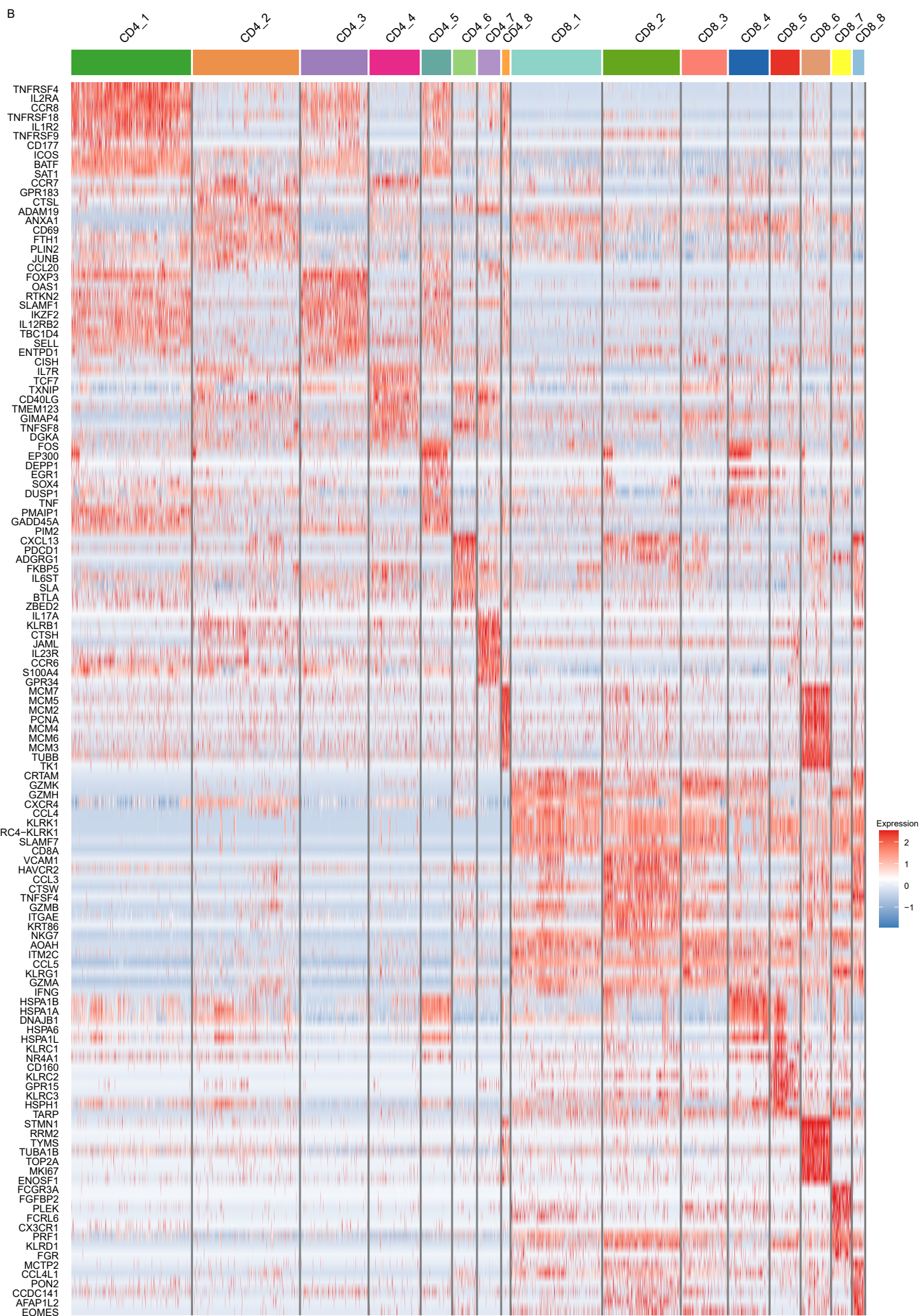

Figure-S5

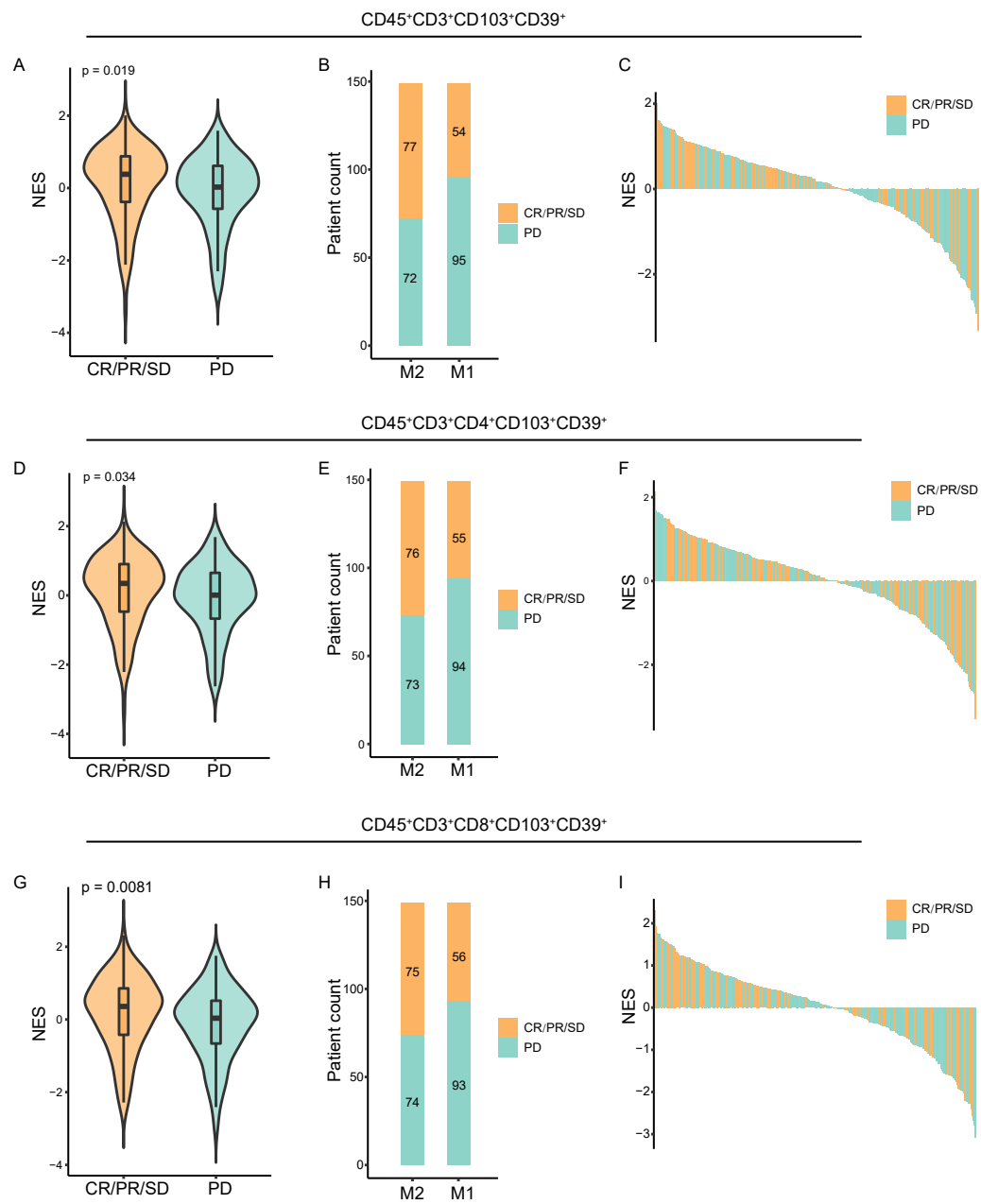

Figure-S6

Supplement: Supplementary Figure 1 — Association between IRScore and clinical factors and molecular signatures. (A). Workflow of this study: IRScore construction and characterization, and IRScore-based detailed immune signatures exploration. (B). Kaplan-Meier curves of recurrence-free survival based on IRScore groups in 7 GEO cohorts. (C). Kaplan-Meier curves of recurrence-free survival based on IRScore groups in a combined dataset. (D, E). Kaplan-Meier curves of disease-specific survival (DSS, D) and overall survival (OS, E) based on IRScore groups in TCGA cohort. (F). Violin plots showing the relationship between IRScore and dMMR/pMMR (left), tumor stages (middle) and Sankey diagram (right) illustrating the relationship between IRScore and CRC subtypes in GSE39582 cohort. (G). The association and correlation between IRScore and different gene signatures (Pan-F-TBRS, naiveness, plasminogen inhibitor, CD8 T effector, cell cycle, cytotoxicity, DNA damage repair, DNA replication, homologous recombination, immune checkpoint, mismatch repair, nucleotide excision repair and exhaustion) in TCGA cohort. [file DataSheet_1.pdf]
